# Diversity, enrichment, and genomic potential of anaerobic methane- and ammonium-oxidizing microorganisms from a brewery wastewater treatment plant

**DOI:** 10.1007/s00253-020-10748-z

**Published:** 2020-06-30

**Authors:** Karin Stultiens, Maartje A.H.J. van Kessel, Jeroen Frank, Peter Fischer, Chris Pelzer, Theo A. van Alen, Boran Kartal, Huub J.M. Op den Camp, Mike S.M. Jetten

**Affiliations:** 1 Department of Microbiology, IWWR, Radboud University Nijmegen, Nijmegen, The Netherlands; 2 Soehngen Institute of Anaerobic Microbiology, Nijmegen, The Netherlands; 3 Present Address: Laboratory of Microbiology, Wageningen University & Research, Wageningen, The Netherlands; 4 MPI Bremen, Bremen, Germany

**Keywords:** Anaerobic oxidation of methane, ‘*Ca*. Methylomirabilis’ bacteria, Anammox, ‘*Ca*. Brocadia’, Nitrite, Metagenome, Hydrazine synthase, pmoA, ‘*Ca.* Methanoperedens nitroreducens’

## Abstract

**Abstract:**

Anaerobic wastewater treatment offers several advantages; however, the effluent of anaerobic digesters still contains high levels of ammonium and dissolved methane that need to be removed before these effluents can be discharged to surface waters. The simultaneous anaerobic removal of methane and ammonium by denitrifying (N-damo) methanotrophs in combination with anaerobic ammonium-oxidizing (anammox) bacteria could be a potential solution to this challenge. After a molecular survey of a wastewater plant treating brewery effluent, indicating the presence of both N-damo and anammox bacteria, we started an anaerobic bioreactor with a continuous supply of methane, ammonium, and nitrite to enrich these anaerobic microorganisms. After 14 months of operation, a stable enrichment culture containing two types of ‘*Candidatus* Methylomirabilis oxyfera’ bacteria and two strains of ‘*Ca*. Brocadia’-like anammox bacteria was achieved. In this community, anammox bacteria converted 80% of the nitrite with ammonium, while ‘*Ca*. Methylomirabilis’ contributed to 20% of the nitrite consumption. The analysis of metagenomic 16S rRNA reads and fluorescence in situ hybridization (FISH) correlated well and showed that, after 14 months, ‘*Ca*. Methylomirabilis’ and anammox bacteria constituted approximately 30 and 20% of the total microbial community. In addition, a substantial part (10%) of the community consisted of *Phycisphaera*-related planctomycetes. Assembly and binning of the metagenomic sequences resulted in high-quality draft genome of two ‘*Ca*. Methylomirabilis’ species containing the marker genes *pmoCAB*, *xoxF*, and *nirS* and putative NO dismutase genes. The anammox draft genomes most closely related to ‘*Ca.* Brocadia fulgida’ included the marker genes *hzsABC*, *hao*, and *hdh*. Whole-reactor and batch anaerobic activity measurements with methane, ammonium, nitrite, and nitrate revealed an average anaerobic methane oxidation rate of 0.12 mmol h^−1^ L^−1^ and ammonium oxidation rate of 0.5 mmol h^−1^ L^−1^. Together, this study describes the enrichment and draft genomes of anaerobic methanotrophs from a brewery wastewater treatment plant, where these organisms together with anammox bacteria can contribute significantly to the removal of methane and ammonium in a more sustainable way.

**Key points:**

• *An enrichment culture containing both N-damo and anammox bacteria was obtained.*

*• Simultaneous consumption of ammonia, nitrite, and methane under anoxic conditions.*

***• ****In-depth metagenomic biodiversity analysis of inoculum and enrichment culture.*

**Electronic supplementary material:**

The online version of this article (10.1007/s00253-020-10748-z) contains supplementary material, which is available to authorized users.

## Introduction

Anaerobic treatment systems offer many advantages such as biogas production in the form of methane and low sludge production. However, the effluents of anaerobic treatment systems still contain dissolved methane and ammonium that need to be removed. While there are several processes that can be used to remove ammonium from the anaerobic effluents, currently there are no established biological methods to remove dissolved methane, resulting in the release of this potent greenhouse gas into the atmosphere (van Kessel et al. 2018; Xie et al. 2018; Liu et al. 2019; Cogert et al. 2019; Fan et al. 2020). For a long time, it was believed that conversion of methane and ammonium required oxygen (reviewed by in’t Zandt et al. 2018). However, in recent years, microbial processes consuming ammonium and methane at the expense of nitrate and/or nitrite have been identified (Strous et al. 1999a; Raghoebarsing et al. 2006; Haroon et al. 2013). The anaerobic ammonium oxidation (anammox) process is performed by bacteria of the *Brocadiales* which use nitrite or nitric oxide as their terminal electron acceptor (Strous et al. 1999a; Hu et al. 2019). Combined with partial nitrification, this process is applied in many full scale municipal and industrial wastewater treatment plants, and has shown to be sustainable system for nitrogen removal (Kartal et al. 2010; Lackner et al. 2014; Hauck et al. 2016). Nitrate- or nitrite-dependent anaerobic methane oxidation (N-damo) by a consortium of methanotrophic bacteria and archaea was first described in 2006 (Raghoebarsing et al. 2006). The N-damo bacteria, named ‘*Candidatus* Methylomirabilis,’ were shown to prefer nitrite as an electron acceptor for methane oxidation, while the N-damo ANME-2d clade archaea seem to use nitrate (Ettwig et al. 2008; Ettwig et al. 2010; Haroon et al. 2013). These archaea, belonging to the genus ‘*Candidatus* Methanoperedens,’ oxidize methane via reverse methanogenesis to carbon dioxide while reducing nitrate via nitrite to ammonium (Haroon et al. 2013; Arshad et al. 2015; Ettwig et al. 2016; Vaksmaa et al. 2017a; Gambelli et al. 2018). Because of this production of both nitrite and ammonium, the ‘*Ca*. Methanoperedens’ archaea are very useful partners for both ‘*Ca*. Methylomirabilis’ and anammox bacteria (Haroon et al. 2013; Shen et al. 2014; Meng et al. 2016; Arshad et al. 2017; Xu et al. 2017; Fu et al. 2019; Nie et al. 2019, 2020). This is supported by studies showing these anaerobic methane- and ammonium-oxidizing microorganisms living together in oxygen-limited ecosystems (reviewed in Welte et al. 2016). In these ecosystems, the organisms may also have to compete with each other under substrate limitation (Luesken et al. 2011a; Winkler et al. 2015; Arshad et al. 2017; Guerrero-Cruz et al. 2019). ‘*Ca*. Methylomirabilis’ and ‘*Ca*. Methanoperedens’ both utilize methane, while ‘*Ca*. Methylomirabilis’ as well as anammox is dependent on nitrite. Oxygen is detrimental for all three processes (Strous et al. 1999b; Luesken et al. 2012; Guerrero-Cruz et al. 2018). Although several studies have enriched combinations of N-damo bacteria and archaea or N-damo archaea and anammox bacteria, few studies have been designed to enrich all three groups from the same source material (Arshad et al. 2017; Stultiens et al. 2019). In order to successfully start such time-consuming long-term enrichment cultures, screening of the inoculum for the presence of markers. Genes of N-damo and anammox microorganisms would be advantageous (Luesken et al. 2011c; Bhattacharjee et al. 2016; Zhu et al. 2017). Previous metagenome analyses have revealed that ‘*Ca*. Methylomirabilis’ uses an intra-aerobic mechanism in which oxygen is produced via a putative nitric oxide dismutase (*nod* gene) and subsequently used for methane oxidation via the particulate methane monooxygenase (*pmo*A gene) complex (Ettwig et al. 2010; Versantvoort et al. 2018). Sequencing of the genome of the ‘*Ca*. Methanoperedens’ indicated the presence of a reverse pathway of methanogenesis via methyl-coenzyme M reductase (*mcr*A), nitrate reductase, and multiheme protein complexes possibly involved in extracellular electron transfer (Ettwig et al. 2010;; Haroon et al. 2013; Arshad et al. 2015; Ettwig et al. 2016; Vaksmaa et al. 2017a; Gambelli et al. 2018). The blueprint of anammox bacteria contains many interesting and diagnostic features including hydrazine synthase (*hzs*A) and hydrazine dehydrogenase, multiheme protein complexes, and nitrite and nitrate reductases (Strous et al. 2006; Kartal et al. 2011).

Using specific primers for (1) anammox 16S rRNA or *hzsA* genes (Schmid et al. 2001; Harhangi et al. 2012); (2) the *pmoA* gene of ‘*Ca*. Methylomirabilis’ (Luesken et al. 2011c); and (3) *mcrA* genes of anaerobic methanogens and methanotrophs (Vaksmaa et al. 2017b), we screened the biomass of a wastewater plant treating brewery effluents for the presence of N-damo and anammox microorganisms. After ‘*Ca*. Methylomirabilis’ and anammox diagnostic genes were detected, we used the biomass to start an anaerobic sequencing batch bioreactor that was continuously fed with nitrate, nitrite, ammonium, and methane. After establishing activity of N-damo and anammox, the total DNA of the new enrichment culture was sequenced, assembled, and binned into draft genomes. The draft genomes of N-damo and anammox were annotated and analyzed. The enriched microbial community was further characterized by fluorescence in situ hybridization (FISH) microscopy and activity assays.

## Materials and methods

### Biomass

In a previous study, 9 WWTP plants in the Netherlands were screened by a nested PCR approach for the presence of the 16S rRNA of N-damo bacteria (Luesken et al. 2011c). One of them, the WWTP in Lieshout (51° 51′ N, 5° 61′ E) treats industrial brewery effluent using UASB reactors followed by a Pasveer-type carrousel at moderate temperatures and intermittent aeration. Sludge from the latter compartment contained the largest diversity of N-damo bacteria. Therefore, biomass of the carrousel sludge of this plant, sampled in November 2013, January 2014, and April 2014, was used for a more extended molecular survey and activity tests. After detection of relevant marker genes of N-damo and anammox microorganisms, 400 mL of the WWTP carrousel sludge (sampled in August 2014) was used as inoculum to enrich for these microorganisms in a continuous bioreactor. The first 3 days of enrichment, oxygen was introduced into the system to mineralize excess sludge; as soon as dissolved oxygen increased, air supply was switched off, and the reactor was operated anoxically by sparging with methane/CO_2_ mixture.

### Batch activity tests

In addition to the molecular survey and to couple these results to physiological tests, we also determined the potential of the Lieshout sludge for aerobic ammonium and nitrite oxidation, denitrification, and anammox activity. Sludge from the carrousel of the Lieshout WWTP was allowed to settle, decanted, and washed 2–3 times with 3-morpholinopropane-1-sulfonic acid (MOPS) buffered N-free mineral salt medium (pH 7.4) to remove excess dissolved organic carbon. Subsequently, the sludge was diluted 6 times with MOPS buffered N-free mineral salt medium (pH 7.4). For all batch incubations, 60 mL of diluted sludge was transferred to 120-mL serum bottles. The headspace of the bottles used for anaerobic incubations (Table 1) was exchanged with argon by 3 cycles of vacuum and gassing followed by 10 min flushing. Next, substrates were added according to Table 1. All treatments were performed in duplicate. Incubations were performed at 30 °C and shaking at 180 rpm. Every 2 h, the availability of nitrite and nitrate was checked by means of Merck test strips, and liquid samples were taken, centrifuged for 1 min at maximum speed, and stored at − 20 °C for further analysis.

**Table 1:** Substrate additions during batch activity assays

Activity	Aerobic/anaerobic	Substrates
Anammox	Anaerobic	2 mM NH_4_Cl + 2 mM NaNO_2_
Aerobic ammonium oxidation	Aerobic	5 mM NH_4_Cl + O_2_
Ammonium oxidation (neg. control)	Anaerobic	5 mM NH_4_Cl
Nitrite oxidation	Aerobic	2 mM NaNO_2_ + O_2_
Nitrite oxidation (neg. control)	Anaerobic	2 mM NaNO_2_
Denitrification	Anaerobic	5 mM NaCH_3_COO + 5 mM NaNO_3_
Denitrification (neg. control)	Anaerobic	5 mM NaNO_3_

### Establishment of an N-damo and anammox enrichment culture

Lieshout carrousel sludge (400 mL) was used to inoculate a 7 L sequencing batch reactor (SBR) containing 4 L medium. Every SBR cycle consisted 10 h and 40 min of constant medium supply, 20 min of settling, and 1-h removal of excess liquid. The first 3-day oxygen was supplied during the filling period to mineralize excess sludge. Dissolved oxygen in the reactor was always below the detection limit of the Clarke-type oxygen sensor (< 0.5 μM). After 3 days, the air supply was turned off, and the culture was kept anoxic by continuous flushing with CH_4_/CO_2_ (95%/5%, 10 mL min^−1^). The culture was fed with synthetic medium (Stultiens et al. 2019) containing sufficient iron, copper, and cerium as trace elements, and was further supplemented with 5 mM NO_3_^−^, 0–8 mM NO_2_^−^, and 0.5–5 mM NH_4_^+^. The NO_2_^−^ and NH_4_^+^ concentration of the medium was gradually increased following the rising consumption of these substrates by the biomass of the culture. The amount of medium fed per day was adjusted to the nitrite and nitrate consumption of the culture. The medium vessel was flushed with Ar/CO_2_ (95%/5%, 10 mL min^−1^). The pH was maintained at 7.3 by KHCO_3_ addition. The reactor was equipped with pH and Clarke-type dissolved oxygen sensors and connected to an ADI1010 bio-controller (Applikon Biotechnology BV, Schiedam, the Netherlands). The SBR was operated a room temperature (20 ± 1 °C) and was stirred by means of a turbine stirrer. Stirring speed was 80 rpm in the first 2 months, after which it was increased to 150 rpm. During the removal period, the liquid volume in the bioreactor was maintained by a level-controlled effluent pump, and washed-out biomass was retained within the system by an external settler. In addition, a gas-buffer bottle (5 L) filled with Argon prevented oxygen from entering the reactor. Influent and effluent samples were taken on a regular basis, centrifuged for 5 min at 20,000×*g* and the supernatant was stored at − 20 °C until further analysis.

### Reactor activity assays

The nitrate, ammonium, nitrite, and methane consumption rates of the anaerobic enrichment culture were determined several times with the whole reactor in batch mode. For the assays that included determination of methane consumption rates, the SBR cycle, influent flow, and CH_4_ inflow were stopped when the reactor level was 4.6–4.7 L. The stirring was stopped and the headspace of the reactor was flushed with Argon. Subsequently, the headspace was closed off and stirring was resumed (150 rpm). Fifty milliliters CO_2_ was added to the headspace, and the gasses in the liquid and headspace were allowed to equilibrate for 18 h. After 18 h, supplements were added as described in Table 2. Gas and liquid samples were taken in regular intervals for further analysis. Anammox activity was evaluated separately in a similar assay. For this assay, after flushing the headspace with Argon for 30 min, the complete reactor was flushed for 2.5 h with Ar/CO_2_ (95%/5%, 10 mL min^−1^). During this flushing period, stirring continued (150 rpm). Subsequently, the headspace was closed off and supplements were added as described in Table 2. Gas and liquid samples were taken in regular intervals for further analysis. Liquid samples taken during these reactor activity tests were centrifuged for 1 min at 20,000×*g* and the supernatant was stored at − 20 °C until further analysis.

**Table 2:** Substrates added in the reactor assays

Reactor assay	Time	Added substrates*
Nitrate and methane consumption	1254	Not applicable*
Nitrate, nitrite, and methane consumption	1265	0.5 mM NO_2_^−^*
Nitrate, nitrite, ammonium, and methane consumption	1268	0.5 mM NO_2_^−^ 0.5 mM NH_4_^+^*
Nitrate, nitrite, and ammonium consumption (anammox)	1271	0.5 mM NO_2_^−^ 0.5 mM NH_4_^+^**

Time, the number of cultivation days

*Methane and nitrate were not added separately but were still present in the liquid of the bioreactor

**Nitrate was not added separately but was still present in the liquid of the bioreactor

## Analytical methods

For reactor samples and samples taken during activity assays, ammonium was determined colorimetrically using a modified orthophataldialdehyde assay and nitrite by the Griess reaction (Ettwig et al. 2008). Nitrate was measured with the NOA280i nitric oxide analyzer (GE Analytical Instruments, Manchester, UK). Nitrate is first converted into nitric oxide at 95 °C using a saturated solution of VCl_3_ in HCl. The produced nitric oxide is subsequently detected and quantified by the nitric oxide analyzer. For samples taken during the reactor activity assays, nitrate was determined in two steps: first, the Griess reaction was performed to determine the nitrite concentration in the samples. Subsequently, the nitrate was reduced to nitrite by adding saturated VCl_3_ solution and incubating the samples for 30 min at 60 °C. The produced nitrite reacted immediately in the Griess assay and gave a combined signal for nitrite and nitrate. Methane was measured using gas chromatography (HP 5890 gas chromatograph with a flame ionization detector, Porapak Q column). N_2_ and N_2_O production was analyzed using gas chromatography (Agilent 6890, Porapak Q column, 80 °C) in combination with mass spectrometry (Agilent 5975c, quadruple inert MS; Ettwig et al. 2010; Arshad et al. 2017).

### Fluorescence in situ hybridization

Biomass samples (2 mL) from the anaerobic methane- and ammonium-oxidizing coculture were taken on day 202 and 408. For each time point, multiple samples (> 5) were processed for FISH analysis. The samples were centrifuged for 5 min at 20,000×*g*. The pellets were washed with 1 mL phosphate-buffered saline (PBS; 10 mM Na_2_HPO_4_/NaH_2_PO_4_ pH 7.5 and 130 mM NaCl) and fixed in paraformaldehyde. Subsequently, hybridization of Cy3, Cy5, or FLUOS fluorescently labeled probes was performed as described before (Arshad et al. 2017; Ettwig et al. 2008). Eub 338, Eub 338 II, and Eub 338 III were mixed in equimolar solution (Eubmix) and used as such. Probes to visualize anammox bacteria (AMX820), ‘*Ca*. Methylomirabilis’ bacteria (DBACT1027), and all Bacteria (Eubmix) were utilized to visualize different groups of microorganisms within the anaerobic methane- and ammonium-oxidizing culture (Amann et al. 1990; Schmid et al. 2001; Raghoebarsing et al. 2006; Daims et al. 1999). All samples were counterstained with DAPI. Slides were examined and images obtained by utilization of a Zeiss Axioplan 2 epifluorescence microscope equipped with a digital camera, in combination with the Axiovision software package (Zeiss,Germany).

### Metagenomic analysis

DNA from all samples was isolated by three different methods: (1) PowerSoil DNA Isolation Kit (MO BIO Laboratories Inc., Carlsbad, CA, USA), (2) the ammonium acetate extraction method (Kowalchuk et al. 2004), and (3) the CTAB extraction method (Zhou et al. 1996). In total, 125 ng of isolated genomic DNA was sheared for 9 min using a Bioruptor® UCD-200 (Thermo Fisher Scientific Inc., USA). Libraries were prepared using an Ion Plus Fragment library kit (Thermo Fisher Scientific Inc., USA). For size selection of the adapter ligated fragments, an E-Gel® electrophoresis system was used with a 2% E-Gel® SizeSelect™ agarose gel (Life Technologies, Bleiswijk, the Netherlands). Eight cycles of amplification of the size-selected fragments were performed. The concentrations and fragment lengths of the libraries were determined with a Bioanalyzer® 2100 and High Sensitivity DNA Kit (Agilent Technologies, Santa Clara, CA, USA). The library was diluted to a final concentration of 26 pM for emulsion PCR. Emulsion PCR was performed using an Ion OneTouch™ 2 Instrument and Ion PGM™ Template OT2 400 Kit (Thermo Fisher Scientific Inc., USA). The template-positive Ion Sphere™ Particles (ISPs) were enriched using the Ion One Touch™ ES and loaded on an Ion 318™ v2 Chip, and sequenced using an Ion PGM™ Sequencing 400 Kit with 850 nucleotide flows. After sequencing, all raw reads were imported into CLC Genomics Workbench version 8.5.1 (QIAGEN Aarhus A/S, Denmark) for initial data analysis, including trimming of low-quality and short reads (cut-off value of 200 nucleotides), followed by assembly of the reads (word size automatic, bubble size 5000). The raw reads of metagenome sequencing are submitted to the Sequence Read Archive (SRA) under project number PRJEB37137. An additional assembly using SPAdes (Bankevich et al. 2012) was performed using standard parameters for single-end reads. All contigs below 1000 bp were discarded from the assembly. To extract the contigs of the anaerobic methanotrophs and anammox, the contigs were binned based on GC content and coverage using RStudio (RStudio Team 2015) with the GC script. The contigs of ‘*Ca.* Methylomirabilis’ or anammox bacteria were extracted from all assemblies, and reads mapping to contigs were reassembled in CLC (word size 30, bubble size 5000). The completeness of the draft genomes and contamination were assessed by CheckM (Parks et al. 2015). BLAST was used to search for key genes in the ‘*Ca.* Methylomirabilis’ and anammox genomes. The contigs containing marker genes were manually curated. The curated contigs were checked using the visualization and annotation tool Artemis (Rutherford et al. 2000).

## Results

### Performance Lieshout WWTP

The carrousel of the WWTP Lieshout is a low-loaded system that receives on average 4700 m^3^ effluent per day from 3 UASB reactors. The total nitrogen supply to the carrousel is about 216 kg N per day and the average ammonium and nitrate concentrations of the UASB effluent are 5.4 and 0.9 mg N L^−1^, respectively. After treatment in the carrousel, the ammonium and nitrate concentrations are 0.7 and 1.2 mg N L^−1^, indicating presence of both nitrifying and denitrifying microorganisms. Therefore, the potential rates for ammonium, nitrite, and nitrate conversions were measured in batch tests (Table 3). The 4.7 mg N L^−1^ ammonium removal in the carrousel results in a removal rate of about 0.33 mmol NH_4_^+^ L^−1^ day^−1^ and is in good agreement with the potential ammonium-oxidation (0.26–0.3 mmol NH_4_^+^ L^−1^ day^−1^) measured in the batch tests. The nitrite-oxidation and nitrate reduction potential exceed these values 2- to 5-fold, indicating that aerobic ammonium oxidation might be the limiting step in the nitrogen removal in WWTP Lieshout. N-damo and anammox activity could not be detected within 36 h of incubation, possibly because of high back ground denitrification rates (Table 3).

**Table 3:** Nitrifying and denitrifying potential of Lieshout sludge

Activity tested	Removal rates (mmol N L^−1^ day^−1^)		
	Nov—2013	Jan—2014	Apr—2014
Ammonium oxidation			
Oxygen-dependent nitrite formation	0.9	1.2	0.5
Oxygen-dependent nitrite + nitrate formation	3.3	4.9	Not determined
Nitrite oxidation			
Oxygen-dependent nitrite removal	2.8	4.1	15
Denitrification			
Anoxic nitrate removal	48	50	46

### Molecular survey Lieshout sludge

**Fig. 1:**
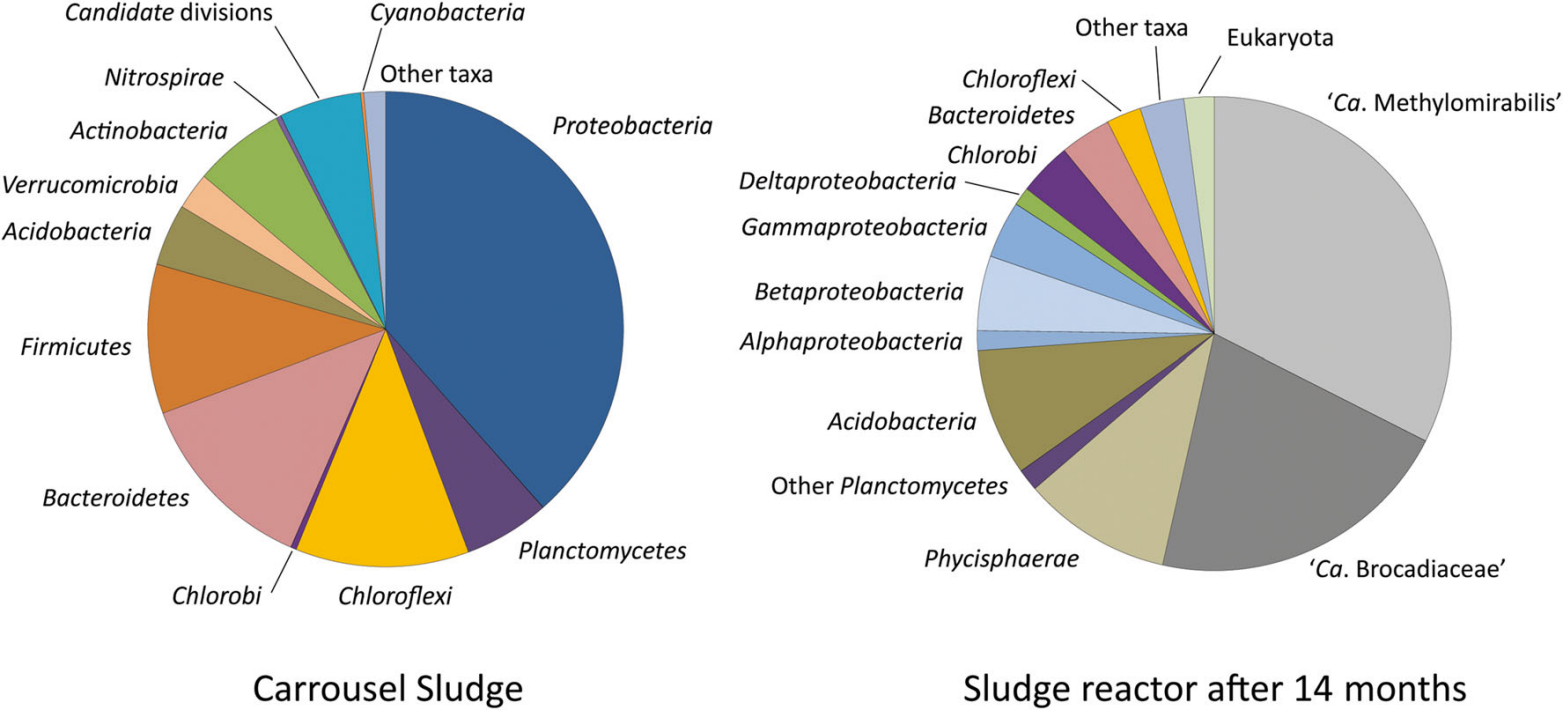
Percentages of reads containing parts of 16S rRNA genes for different phyla extracted from the metagenomes of **a** the original Lieshout WWTP sludge and **b** the enrichment bioreactor metagenome after 14 months of operation, by mapping on the Silva database

DNA of the Lieshout sludge was extracted and sequenced using Ion Torrent Technology. Extraction of 16S rRNA gene sequences (Fig. 1a) of the total metagenome yielded 2801 reads, the majority belonging to *Proteobacteria* (38%), *Bacteroidetes* (13%), *Chloroflexi* (12%), and *Firmicutes* (10%). *Planctomycetes* comprised 6% of the 16S rRNA reads, of which many corresponded to the order of *Phycisphaerales*. In the metagenome, no 16S rRNA reads belonging to anammox bacteria were retrieved, while 23 (0.8%) reads were affiliated to *Nitrosomonas* and 25 (0.9%) to aerobic methanotrophs (*Methylococcocaea*). Despite the absence of anammox reads in the metagenome, 16S rRNA and hydrazine synthase (*hzs*A) genes could readily be amplified using DNA from the Lieshout carrousel sludge as a template. The majority of anammox clones were affiliated to ‘*Ca*. Brocadia’ (Supplementary Fig. S1). Also, the *pmo*A gene of ‘*Ca*. Methylomirabilis’ could be amplified from the sludge DNA, and the diversity of these N-damo bacteria (Supplementary Fig. S2) was similar to previous studies based on nested 16S rRNA gene amplifications (Luesken et al. 2011c). The amplification of the *mcr*A gene showed a large diversity of methanogens (Supplementary Fig. S3) probably seeded into the carrousel from the UASB effluent. One of the 16 analyzed *mcr*A clones was affiliated to N-damo ‘*Ca*. Methanoperedens’ archaea.

### Enrichment of N-damo and anammox bacteria

**Fig. 2:**
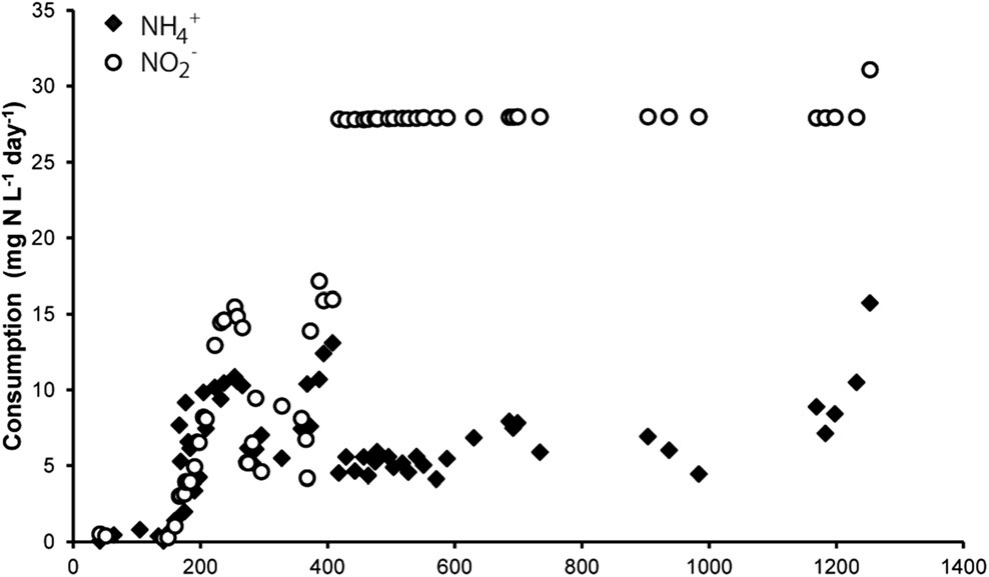
Conversion of ammonium and nitrite in the enrichment of N-damo and anammox from WWTP Lieshout

**Fig. 3:**
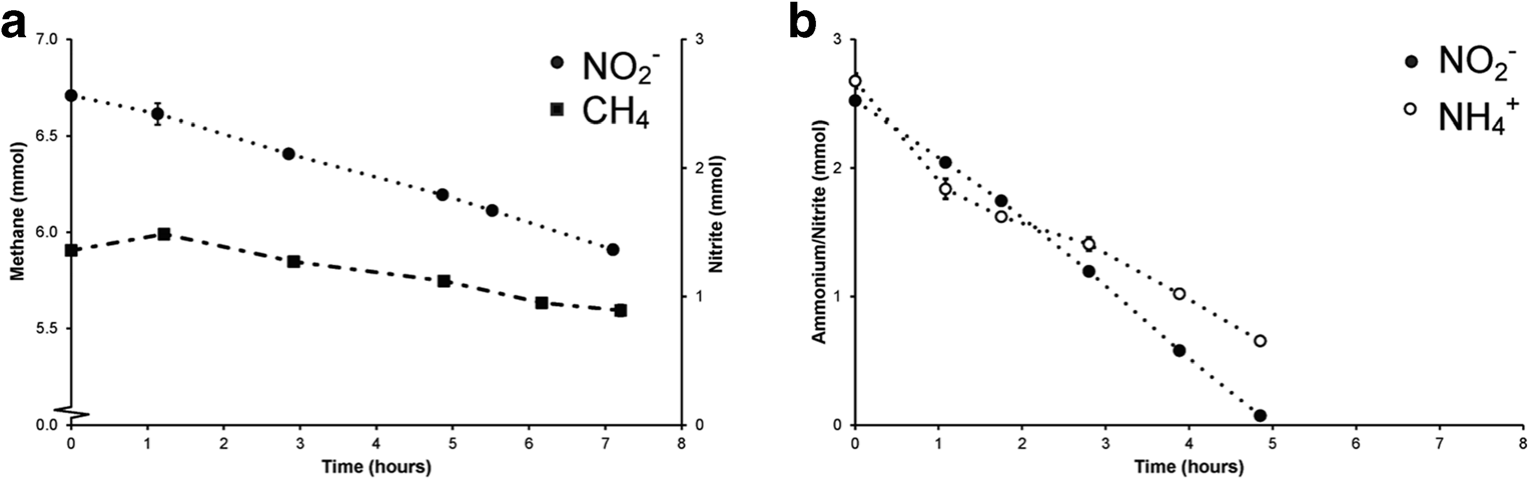
**a** Conversion of methane and nitrite by N-damo. **b** Conversion of ammonium and nitrite by anammox in the Lieshout N-damo and anammox enrichment culture after roughly 42 months of operation

As anammox and N-damo bacteria were present in relatively low abundance, the excess biomass in the sludge needed to be mineralized as a strategy to reduce enrichment times. The first 3 days after inoculation of an anaerobic SBR, air was introduced into the system to convert easily degradable carbon and biomass. After the dissolved oxygen concentration started to increase, the air supply was turned off, and the SBR was fed with methane, ammonium, nitrate, and nitrite under anoxic conditions. After 100 days, the N-load to the reactor could gradually be increased to 5 mM NH_4_^+^, 8 mM NO_2_^−^, and 5 mM NO_3_^−^ resulting in nitrite and ammonium consumption rates of 1.25 mmol L^−1^ day^−1^ and 1 mmol L^−^1 day^−1^, respectively (Fig. 2). When the reactor was run in batch mode without ammonium present, nitrite consumption (0.12 mmol h^−1^) at the expense of methane could readily be observed (Fig. 3a). In the absence of methane, anammox bacteria converted nitrite (0.5 mmol h^−1^) with ammonium as electron donor (Fig. 3b), indicating that anammox converted about 80% of the nitrite fed to the reactor while N-damo was responsible for the remaining 20%.

**Fig. 4:**
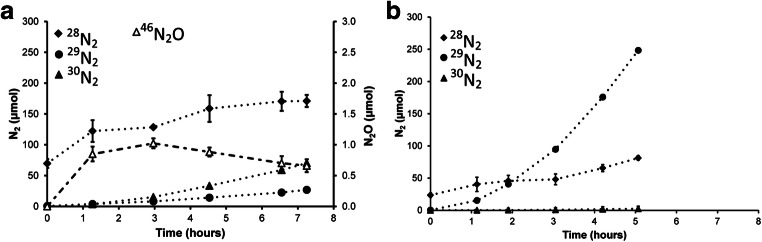
**a** Labeled dinitrogen gas production from ^15^N nitrite with methane as electron donor by N-damo, without presence of ammonium. **b** Labeled dinitrogen gas production from ^15^N ammonium with nitrite by anammox, without methane present, in the Lieshout N-damo and anammox enrichment culture after 42 months of operation

The fate of ^15^N-labeled substrates was measured in a reactor batch test. According to expectation (Ettwig et al. 2010), labeled ^15^N-nitrite was predominantly converted to ^30^N_2_ gas (Fig. 4a) by the N-damo bacteria. In the anammox test, most of the ^15^N label of ammonium was converted in to ^29^N_2_ following the described stoichiometry (Strous et al. 1999b). In both incubation, some ^28^N_2_ from unlabeled nitrate was produced presumably via background denitrification, and therefore exact mass balanced could not be made.

**Fig. 5:**
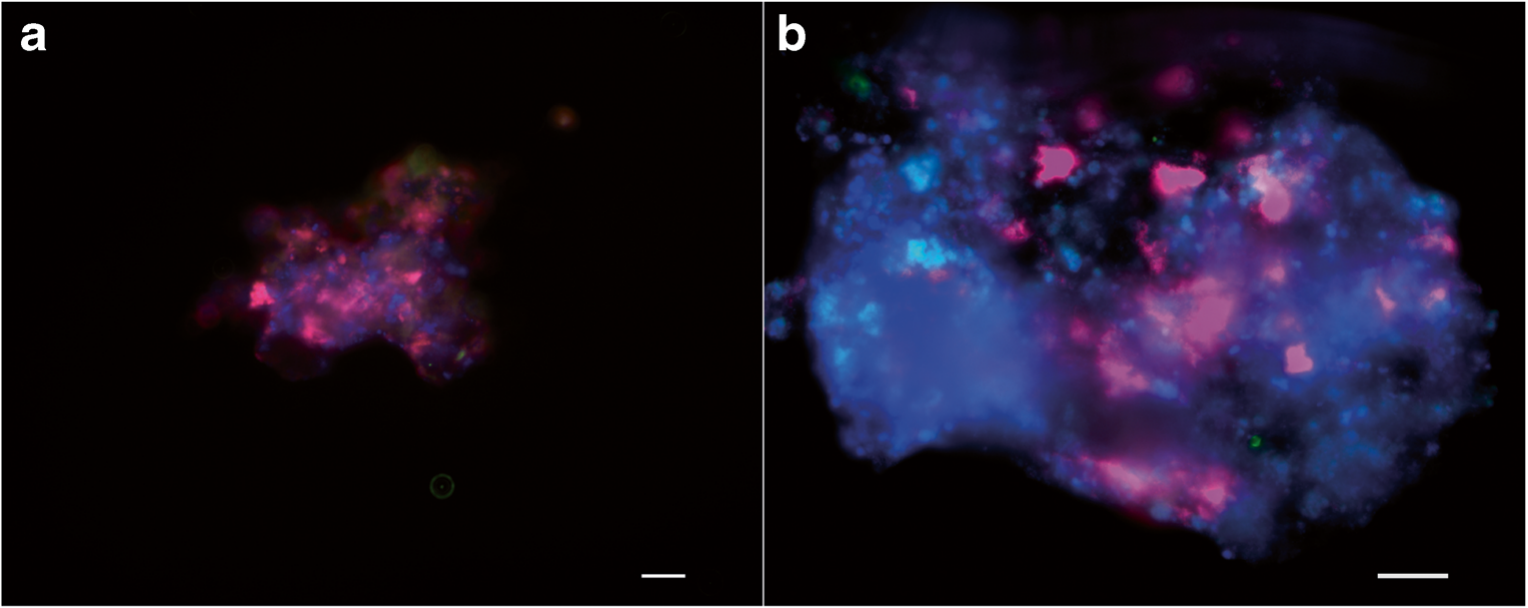
FISH microscopy of the N-damo and anammox enrichment. **a** After 200 days, anammox bacteria were shown to be present (amx820, pink) whereas N-damo bacteria could not be visualized by FISH (damo1027, green). **b** After 408 days of enrichment, both anammox bacteria (amx820, blue) and N-damo bacteria (damo1027, pink) were clearly present. Scale bar represents 20 μm

In accordance with the nitrogen conversions in reactor batch tests, ‘*Ca*. Methylomirabilis’ and anammox bacteria were the dominant members of the community as estimated from FISH microscopy (Fig. 5) and metagenomic analysis after 14 months of operation.

### Metagenomic analysis and classification based on the 16S rRNA gene

After 4.5 months of enrichment, genomic DNA was extracted and sequenced by Ion Torrent Technology to investigate enrichment of anaerobic ammonia and methane-oxidizing microorganisms. Not much changes were observed compared with the original sludge and we decided to continue the enrichment. After 14 months of enrichment, DNA was extracted once more to analyze the 16S rRNA gene diversity and to potentially assemble draft genomes. From this metagenome, 1996 16S rRNA reads were extracted. The phylogenetic classifications for groups with an abundance of greater than 1% of the total number of 16S rRNA gene reads are shown in Fig. 1b. More than 60% of the 16S rRNA gene reads belonged to three dominant groups: *Brocadiaceae* (21%), N-damo ‘*Ca*. Methylomirabilis’ bacteria (32%), and *Phycisphaera*-like *Planctomycetes* (10%), confirming the results of FISH microscopy.

The Ion Torrent reads were assembled into contigs, and contigs were binned by differential coverage and GC content. The binning resulted in 6 high-quality draft genomes (above 92% completeness; Table 4). Two draft genomes (Bins 1 and 2) represented distinct ‘*Ca*. Methylomirabilis’ species, closely related to ‘*Ca.* M. oxyfera’ (Bin2) and ‘*Ca.* M. lanthanidiphila’ (Bin1). Three bins showed high similarity to ‘*Ca*. Brocadia’ anammox species and further refinement was hampered by microdiversity differences. Interestingly, one draft genome (Bin6) represented an unknown genus, most closely related to the *Phycisphaera* species within the phylum *Planctomycetes*.

**Table 4:** Overview of bin quality, completeness, and characteristics

Bin	Identity	Completeness (%)	Contamination (%)	Number of contigs	Genome size (bp)	Assembly	Binning methods
1	‘*Ca*. Methylomirabilis’	93.3	6.8	67	3,073,612	SPAdes	Metabat
2	‘*Ca*. Methylomirabilis’	95.4	1.7	60	2,866,557	CLC	Metabat, reassembly
3	‘*Ca*. Brocadia’	93.3	0	145	3,129,904	CLC	Metabat, manual, differential
5	‘*Ca*. Brocadia’	92.2	8.7	224	3,753,638	CLC	Metabat, manual, differential
6	‘*Phycisphaera*’	94.0	0	74	5,039,581	SPAdes	Metabat
8	‘*Ca*. Brocadia’	95.6	1.7	174	4,185,325	CLC	Metabat, manual, differential

The ‘*Ca*. Methylomirabilis‘ draft genomes (Bins 1 and 2) contained the diagnostic *pmoCAB*, *xoxF*, *nirS*, and putative NO dismutase genes. The *pmoA* marker genes encoding a subunit of the membrane-bound methane monooxygenase had 99.2% and 97.1% identity at the protein level to ‘*Ca*. M. oxyfera’ and ‘*Ca.* M. lanthanidiphila,’ respectively. We also identified a gene encoding the lanthanide-dependent methanol dehydrogenase (*xoxF*) in both genomes with 93–98% identity at the protein level to ‘*Ca*. Methylomirabilis’ species. Genes encoding the canonical calcium-dependent methanol dehydrogenase were absent in both genomes. Genes encoding the putative nitric oxide dismutase (*nod*) showed 85–90% amino acid identity to ‘*Ca*. Methylomirabilis’ species. The identified cd1 cytochrome nitrite reductase (*nirS*) had 92–99% identity to other ‘*Ca*. Methylomirabilis’ species.

The genomes assembled in Bins 3, 5, and 8 were determined to belong to the Planctomycetes phylum by the CheckM taxonomy assessment and most closely related to the anammox bacterium ‘*Ca*. Brocadia fulgida.’ This points to microdiversity of this species with bin3 as the most dominant (Supplementary Fig. S4). The genes encoding the crucial proteins involved in the anammox process were identified in the bins. These proteins include the hydrazine synthase, the hydrazine dehydrogenase, and multiple hydroxylamine oxidoreductases. The phylogenetic functional marker genes *hzsA* (Harhangi et al. 2012) encoding the alpha subunit of the hydrazine synthase had 89–93% identity on protein level to ‘*Ca*. Brocadia’ species.

The genome present in Bin6 was the only bin with complete 16S rRNA and 23S rRNA genes on one of the contigs. A BlastN search with the 16S rRNA gene revealed 99.3% identity with clones from anammox reactors treating wastewater (NCBI, unpublished sequences). The closest cultured representative with 92.4% identity was *Phycisphaerae* bacterium RAS2 isolated from a fish tank biofilter enrichment (Wiegand et al. 2020). The Prokka annotated genome does not reveal the potential for an autotrophic life style.

## Discussion

Nitrate- and nitrite-dependent anaerobic oxidation of methane (N-damo) was discovered a decade ago, but the ecophysiological characterization of the microorganisms involved has been hindered by the slow growth of the responsible organisms (Raghoebarsing et al. 2006; Ettwig et al. 2010; Haroon et al. 2013). The N-damo microorganisms ‘*Candidatus* Methanoperedens nitroreducens’ archaea and ‘*Ca*. Methylomirabilis’ bacteria have been detected in various freshwater sediments (Welte et al. 2016). In this study, we started an enrichment culture fed with methane, ammonium, nitrate, and nitrite using carrousel sludge from a WWTP treating brewery effluent, as the inoculum. Based on activity measurements, FISH microscopy, and metagenome analyses after 14 months, the enrichment was dominated by ‘*Ca*. Methylomirabilis’ species and anammox bacteria. Previous enrichments were fed with mixtures of nitrite and nitrate without ammonium, to ensure that anammox bacteria would not outcompete ‘*Ca*. Methylomirabilis’ bacteria for nitrite (Luesken et al. 2011b; Shi et al. 2013; Hu et al. 2015; Ding et al. 2017; Vaksmaa et al. 2017b). Although the inoculum sludge had no detectable anammox or N-damo activity within 36 h, and only one 16S rRNA gene read resembling anammox could be detected in the initial metagenome, the diagnostic genes *hzsA* and *pmoA* could be readily amplified using DNA extracted from the sludge as a template. This indicated that the Lieshout carrousel sludge does harbor indigenous populations of N-damo and anammox bacteria and could serve as start-up material when large-scale installation need to be seeded (van Kessel et al. 2018). Indeed, after a start-up phase of about 100 days, an increasing consumption of nitrite, methane, and ammonium could be observed, and ultimately anammox and N-damo bacteria were the key players of the microbial community. The start-up phase was relatively short, as previous enrichments obtained from a minerotrophic peatland only showed significant methane oxidation rates after 9 months and the original enrichment of N-damo took more than a year before activity could be measured (Raghoebarsing et al. 2006; Zhu et al. 2012). In addition to substrate preference and availability, temperature has been implicated as a decisive factor in the outcome of N-damo enrichments. In enrichments started from wastewater treatment sludge and lake sediments, a co-enrichment of ‘*Ca*. Methylomirabilis’ bacteria and ‘*Ca*. Methanoperedens’ archaea was obtained at 35 °C, whereas at 22 °C, only N-damo bacteria were enriched (Hu et al. 2009). Furthermore, ‘*Ca*. Methanoperedens’ might be more sensitive to oxygen exposure (Guerrero-Cruz et al. 2018) than anammox and ‘*Ca*. Methylomirabilis’ bacteria (Luesken et al. 2011a, 2012). As we used air in the first 3 days of enrichment to degrade excess sludge, we may have strongly inhibited the few ‘*Ca*. Methanoperedens’ cells present and prevented their subsequent enrichment.

In the present enrichment, the cell numbers estimated by FISH correlated well with the metagenome sequencing results for ‘*Ca*. Methylomirabilis,’ with an abundance of about 30–40%. Anammox seemed somewhat underrepresented in the metagenome (20%) as activity measurements indicated that 80% of the nitrite conversion was accounted for by anammox activity. A similar observation was made previously where anammox converted about 70% of nitrite while FISH counts of anammox were less than 50% of the community (Luesken et al. 2011a). In our enrichment, there was always ammonium-limiting to ensure that ‘*Ca*. Methylomirabilis’ was not outcompeted by anammox (Hu et al. 2015). Incubations performed with the whole bioreactor revealed average ammonium oxidation and nitrite reduction after 14 months of 1 mmol day^−1^ L^−1^ and 1.25 mmol day^−1^ L^−1^, respectively which is a little bit lower compared with other enrichments (Luesken et al. 2011b; Hu et al. 2009; Vaksmaa et al. 2017b). With ^15^N isotope labeling experiments, we could show that in the absence of ammonium, a good methane-dependent nitrite conversion took place, and that the dominant product was the expected ^30^N_2_ gas (Ettwig et al. 2010). In the absence of methane, ^15^N-labeled ammonium was combined with ^14^N nitrite to form ^29^N_2_ by anammox bacteria in accordance with the anammox reaction equation (Strous et al. 1999b). As not all unlabeled nitrate could be removed from the reactor, some background denitrification and N_2_O production was observed as well, as has been reported for other N-damo enrichments (Vaksmaa et al. 2017b).

Metagenome analysis revealed that ‘*Ca.* Methylomirabilis,’ ‘*Ca*. Brocadia,’ and a *Phycisphaera*-like bacterium were the dominant members of the community after 14 months of enrichments. The diagnostic genes for nitrite-driven anaerobic ammonium and methane oxidation were identified in the genomes assembled from the metagenome sequences. To elucidate the role of the *Phycisphaera*-like bacterium, further research will be needed. Recently, members of the family *Phycisphaeraceae* have been reported to be potentially involved in anaerobic ammonium oxidation coupled to the reduction of sulfate (Sulfammox) or Fe(III) reduction (Feammox) in marine sediments (Rios-Del Toro et al. 2018a). Furthermore, representatives of this family were dominant in marine sediments from the eastern tropical North Pacific coast (Rios-Del Toro et al. 2018b). In this study, anaerobic ammonium oxidation linked to the microbial reduction of natural organic matter (NOM) fueled nitrogen loss. Other community members in our reactor system, mostly less than 5% of the population, belonged to various groups that have been reported before to be present in anaerobic methanotrophic enrichments (Vaksmaa et al. 2017b). In the enrichment, betaproteobacterial *Rhodocyclaceae* accounted for 6% and *Comamonadaceae* for 4% of the genome reads. They have both been implicated to perform denitrification, which may explain the observed back ground nitrate reduction, which was higher than expected based on the methane oxidation rate alone. Further significant numbers of *Chloroflexi* were present. They are obligate anaerobes that have previously been found in both anaerobic methanotrophic (Ettwig et al. 2009; Siniscalchi et al. 2015) and methanogenic enrichment cultures (Yamada et al. 2005; Gray et al. 2011; Liang et al. 2015). These *Chloroflexi* may degrade organic acids to formate, acetate, and hydrogen and feed the other community members (Hug et al. 2013).

In summary, we have enriched a N-damo and anammox coculture from the Lieshout WWTP carrousel sludge. Metagenome analysis, FISH microscopy, and activity test were in good agreement with each other that anammox contributed most to the nitrite conversion under ammonium limitation. The newly enriched co-culture will be used in future studies to unravel the ecophysiological properties of N-damo and anammox under oxygen limitation and investigate their potential role in more sustainable wastewater treatment systems.

## Electronic supplementary material

ESM 1 (PDF 147 kb)

## Data Availability

The raw reads of the metagenome sequencing are submitted to the Sequence Read Archive (SRA) under project number PRJEB37137.
