# Supplementary material for: Diversity, enrichment, and genomic potential of anaerobic methane- and ammonium-oxidizing microorganisms from a brewery wastewater treatment plant
Source: Appl Microbiol Biotechnol. 2020 Jun 30;104(16):7201–12. doi: 10.1007/s00253-020-10748-z (PMC7374466; doi:10.1007/s00253-020-10748-z)
Supplement: Supplementary file 1 — (PDF 147 kb) [file 253_2020_10748_MOESM1_ESM.pdf]

Applied Microbiology and Biotechnology

Supplementary Material

Diversity, enrichment and genomic potential of anaerobic methane- and ammonium-oxidizing microorganisms from a brewery wastewater treatment plant

Karin Stultiens[1], Maartje A.H.J. van Kessel[1,2], Jeroen Frank[1], Peter Fischer[1,2$], Chris Pelzer[1], Theo A. van Alen[1], Boran Kartal[1,3], Huub J.M. Op den Camp[1*], Mike S.M. Jetten[1,2]

[1] Department of Microbiology, IWWR, Radboud University Nijmegen, Nijmegen, the Netherlands

[2] Soehngen Institute of Anaerobic Microbiology, Nijmegen, the Netherlands

[3] MPI Bremen, Germany

[$] Present address: Laboratory of Microbiology, Wageningen University & Research, the Netherlands

*Address correspondence to: Huub J.M. Op den Camp, h.opdencamp@science.ru.nl, +31 243652657

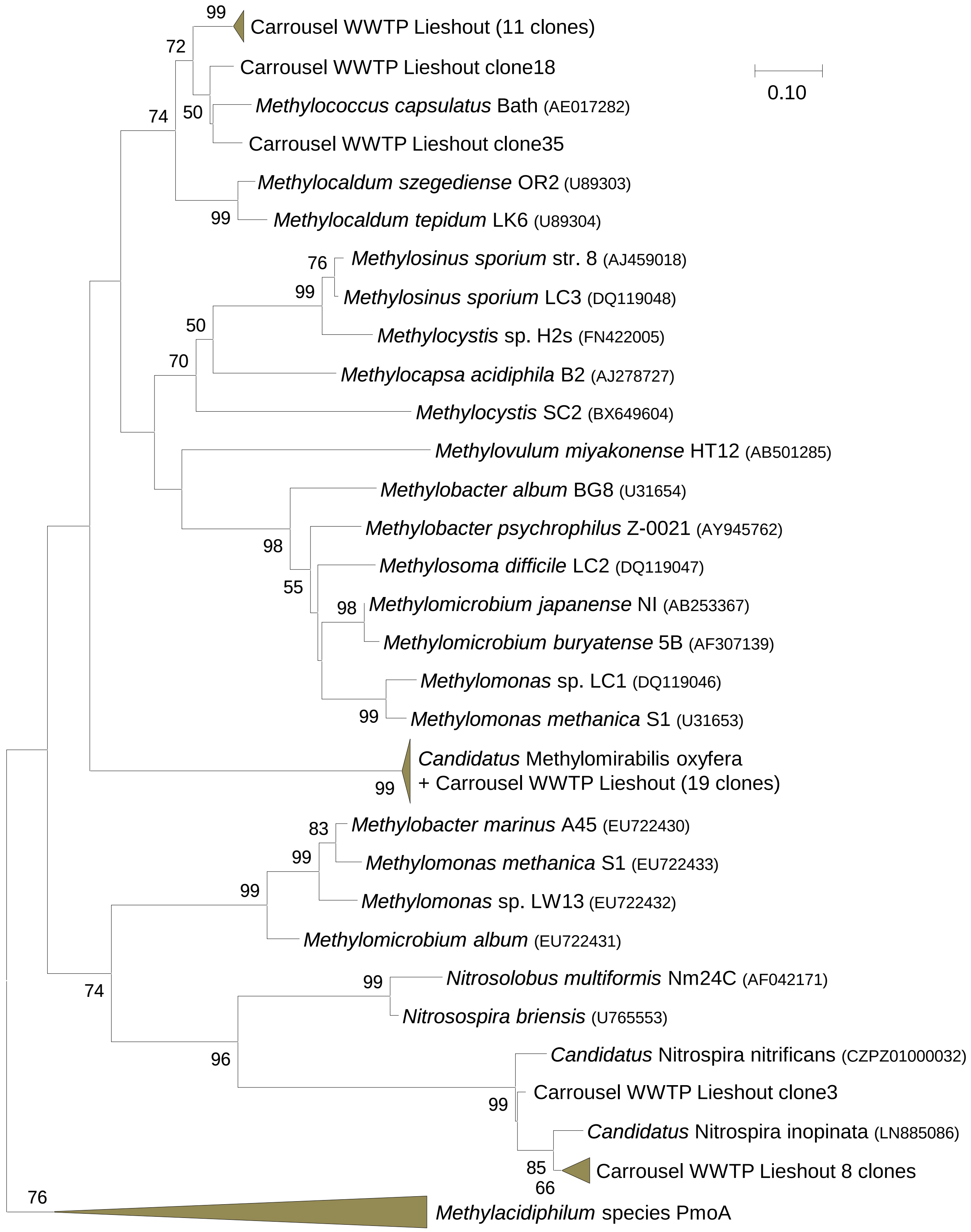

Supplementary Fig. S1. Neighbour-Joining phylogenetic tree of PmoA protein sequences. The optimal tree with the sum of branch length = 6.85353390 is shown. The percentage of replicate trees in which the associated taxa clustered together in the bootstrap test (500 replicates) are shown next to the branches. The tree is drawn to scale, with branch lengths in the same units as those of the evolutionary distances (Dayhoff matrix) used to infer the phylogenetic tree. The evolutionary distances are in the units of the number of amino acid substitutions per site. The analysis involved 75 amino acid sequences. All ambiguous positions were removed for each sequence pair. There were a total of 117 positions in the final dataset. Evolutionary analyses were conducted in MEGA7.

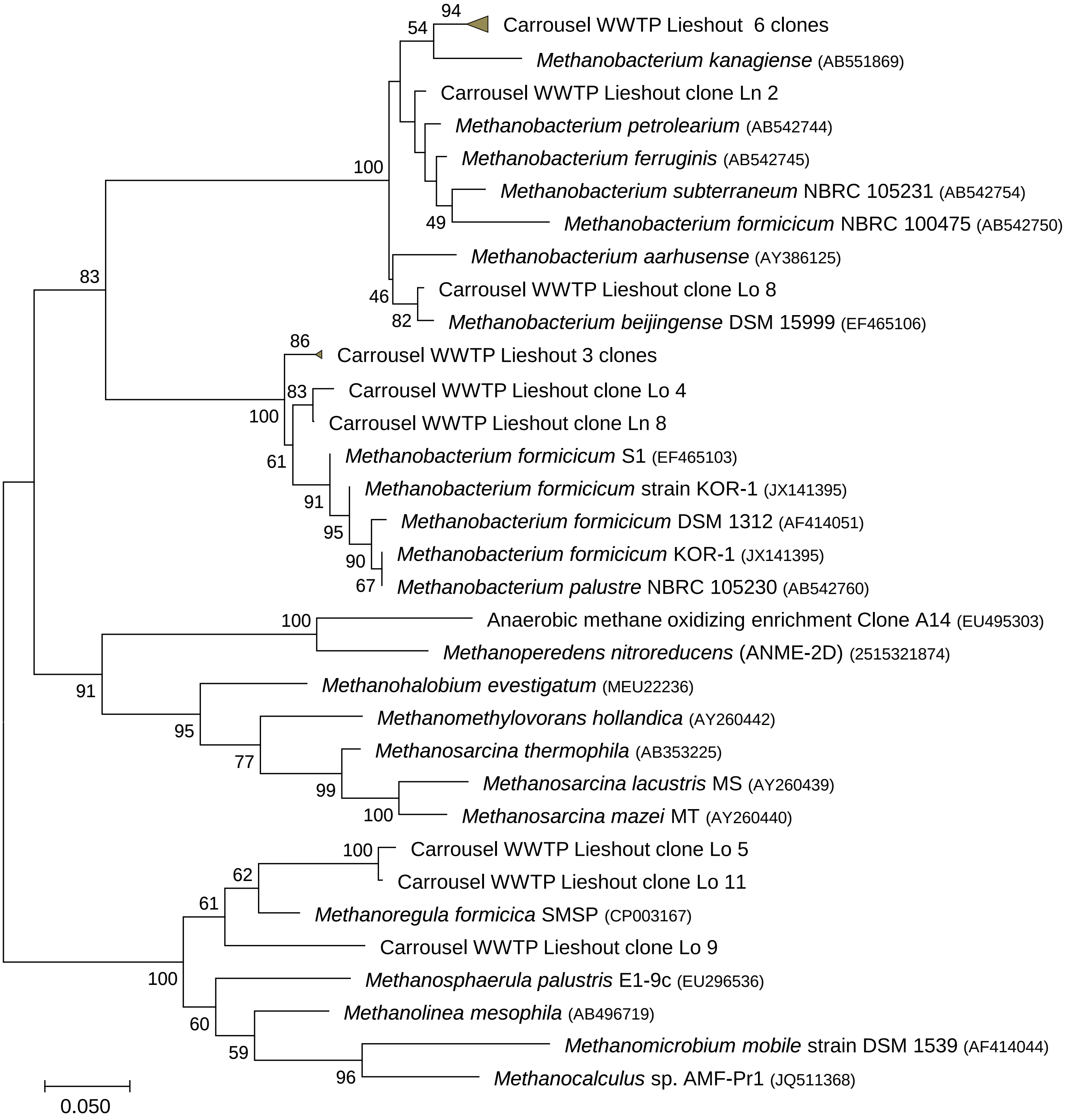

Supplementary Fig. S2. Neighbour-Joining phylogenetic tree of McrA protein sequences. The optimal tree with the sum of branch length = 2.22930066 is shown. The percentage of replicate trees in which the associated taxa clustered together in the bootstrap test (500 replicates) are shown next to the branches. The tree is drawn to scale, with branch lengths in the same units as those of the evolutionary distances (Dayhoff matrix) used to infer the phylogenetic tree. The evolutionary distances are in the units of the number of amino acid substitutions per site. The analysis involved 40 amino acid sequences. All ambiguous positions were removed for each sequence pair. There were a total of 573 positions in the final dataset. Evolutionary analyses were conducted in MEGA7.

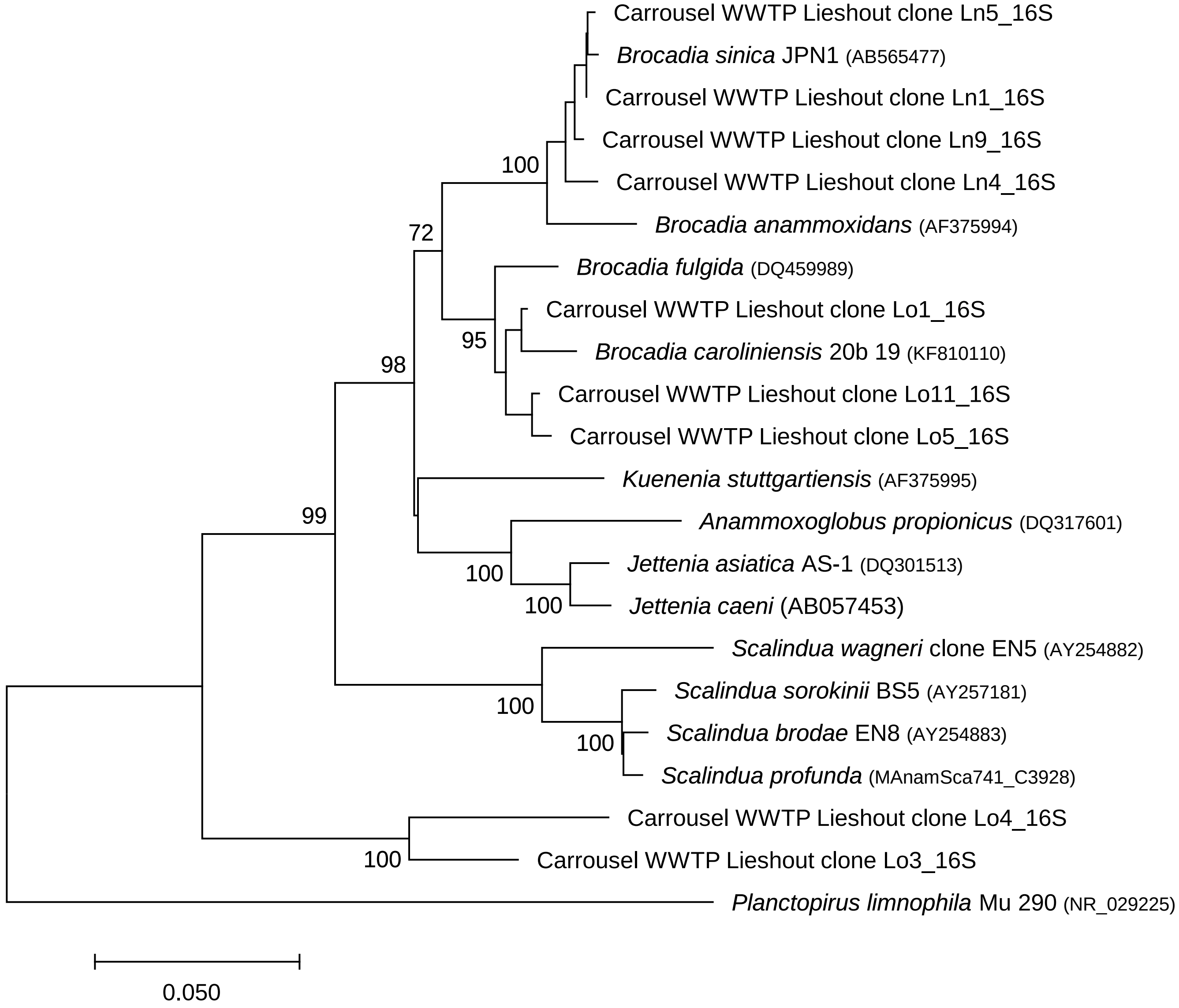

Supplementary Fig. S3. Neighbour-Joining phylogenetic tree of anammox-specific 16S ribosomal RNA genes. The optimal tree with the sum of branch length = 0.81351381 is shown. The percentage of replicate trees in which the associated taxa clustered together in the bootstrap test (500 replicates) are shown next to the branches. The tree is drawn to scale, with branch lengths in the same units as those of the evolutionary distances used to infer the phylogenetic tree. The evolutionary distances were computed using the Jukes-Cantor method and are in the units of the number of base substitutions per site. The analysis involved 22 nucleotide sequences. All ambiguous positions were removed for each sequence pair. There were a total of 1567 positions in the final dataset. Evolutionary analyses were conducted in MEGA7.

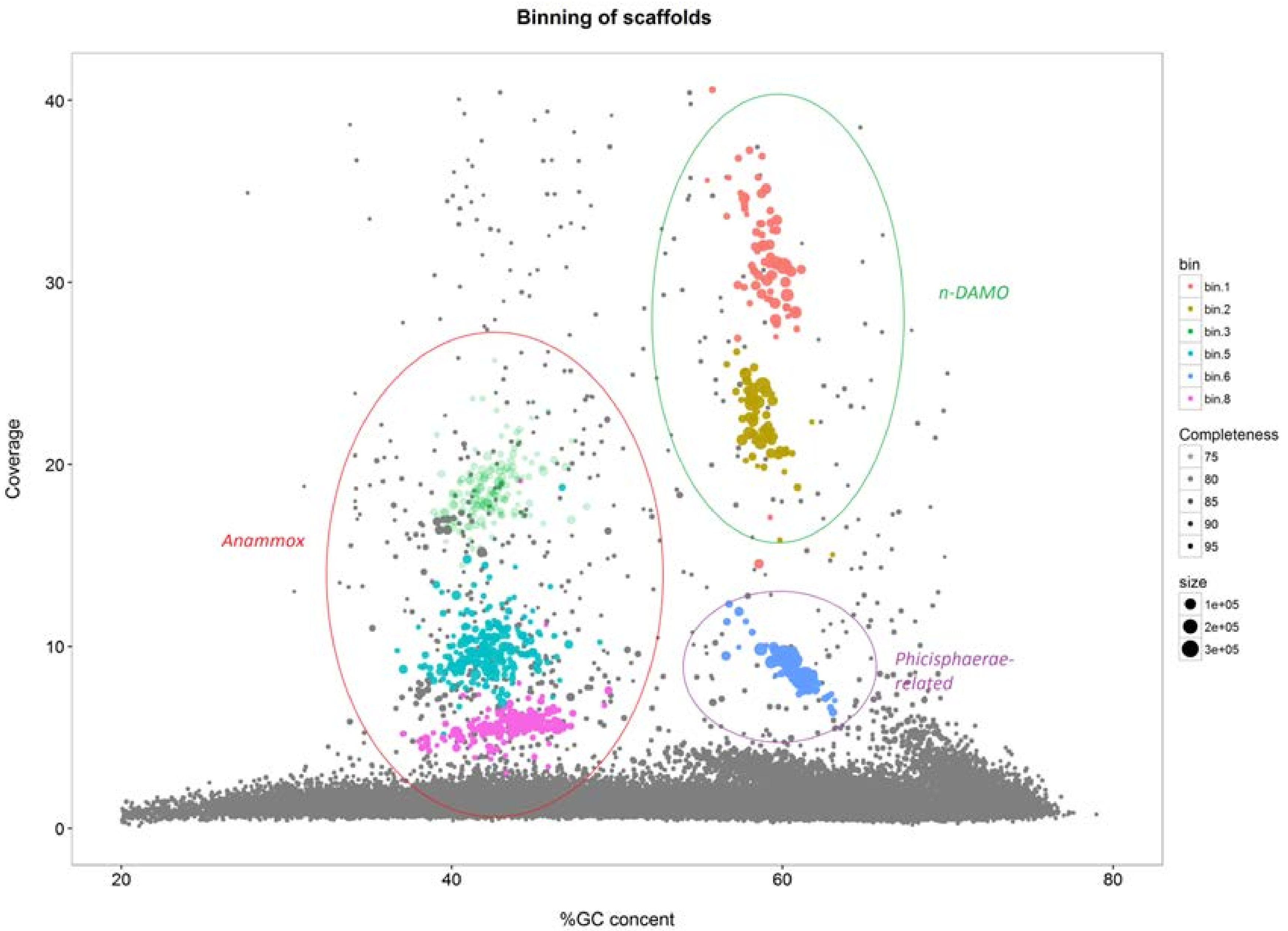

Supplementary Fig. S4. Visualization of reactor metagenome assembly and binning . Contigs are plotted based on GC identity and coverage. Bins representing the same guild are circled in green (‘Ca. Methylomirabilis’, n-DAMO), red (‘Ca. Brocadia’, anammox) and purple (unknown Planctomycete).
